# Supplementary material for: Deep Phenotypic Analysis of Blood and Lymphoid T and NK Cells From HIV+ Controllers and ART-Suppressed Individuals
Source: Front Immunol. 2022 Jan 27;13:803417. doi: 10.3389/fimmu.2022.803417 (PMC8829545; doi:10.3389/fimmu.2022.803417)
Supplement: Supplementary file 1 [file DataSheet_1.pdf]

Supplementary Material

1 Supplementary Tables

1.1 Table S1. Participant characteristics.

Table S1. Participant characteristics. The age, gender, race/ethnicity, antiretroviral regimen (ART), viral load (VL) levels in copies/ml, absolute CD4 T cell count, and absolute CD8 T cell count of each participant at time of specimen collection are indicated.

PID	Category	Age	Gender	Race/Ethnicity	ART	VL	CD4 Count	CD8 Count
1349*	Controller	63	Male	White	None	<40	573	1042
1508*	Controller	56	Male	Black/African American	None	81	425	1394
1559*	Controller	60	Male	Black/African American	None	<40	491	442
1775*	Controller	39	Male	White	None	776	851	882
3736	Controller	60	Male	White	None	<40	853	406
8007*	Acutely-treated	66	Male	White	BIC/FTC/TAF	<40	702	646
8009*	Acutely-treated	29	Male	Asian	ABC/TCV/3TC	<40	547	444
8011*	Acutely-treated	46	Male	Hispanic/Latino	BIC/FTC/TAF	<40	738	386
8012	Acutely-treated	49	Male	White	TCV, TRU	<40	478	471
8019*	Acutely-treated	31	Male	White	RPV/TAF/FTC, TCV	<40	448	354
8039	Acutely-treated	24	Male	Mixed Race/ Multiracial	ABC/TCV/3TC	<40	405	598
8053*	Acutely-treated	35	Male	Mixed Race/ Multiracial	BIC/FTC/TAF	<40	516	1059
8083	Acutely-treated	27	Male	Mixed Race/ Multiracial	BIC/FTC/TAF	108	166	319
1379*	Chronically-treated	50	Male	Black/African American	DRV, RTV, TRU	<40	522	596
2021	Chronically-treated	52	Male	Black/African American	RPV/TAF/FTC	<40	500	857
2161*	Chronically-treated	71	Male	White	DRV, RTV, TCV, 3TC	<40	568	479
2253	Chronically-treated	69	Male	White	BIC/FTC/TAF	<40	349	354
2553*	Chronically-treated	36	Female	Hispanic/Latino	ABC/TCV/3TC	<40	562	721
3037*	Chronically-treated	61	Male	Black/African American	BIC/FTC/TAF, DRV, DOR	54	598	1326

Abbreviations: BIC: Bictegravir; FTC: Emtricitabine; TAF: Tenofovir alafenamide; ABC: abacavir; TCV: Tivicay; 3TC: lamivudine; TRU: Truvada; RPV: Rilpivirine; DRV: Darunavir; RTV: Ritonavir; DOR: Doravirine. *Indicates those participants from which paired blood and lymph node specimens were collected.

1.2 Table S2. List of CyTOF antibodies used in this study. Antibodies were either purchased from the indicated vendor, or prepared in-house using commercially available MaxPAR conjugation kits according to the manufacturer's instructions (Fluidigm).

Antigen Target	Clone	Elemental Isotope	Vendor
CD57	HNK-1	89Y	In-house
HLA-DR	Tu36 / Q22158	Qdot (112Cd)	ThermoFisher
RORγt*	AFKJS-9	115In	In-house
CD25	M-A251	141Pr	In-house
CD19	HIB19	142Nd	Fluidigm
CD14	M5E2	142Nd	In-house
CD33	WM53	142Nd	In-house
CD94	DX22	143Nd	In-house
CCR5	NP6G4	144Nd	Fluidigm
Siglec-7	S7.7	145Nd	In-house
CD8	RPAT8	146Nd	Fluidigm
CD7	CD76B7	147Sm	Fluidigm
ICOS	C398.4A	148Nd	Fluidigm
Tbet*	4B10	149Sm	In-house
NKp30	P30-15	150Nd	In-house
NKp46	9E2	151Eu	In-house
TCRγ/δ	11F2	152Sm	Fluidigm
CD62L	DREG56	153Eu	Fluidigm
TIGIT	MBSA43	154Sm	Fluidigm
CCR6	11A9	155Gd	In-house
NKG2D	1D11	156Gd	In-house
KIR2DL1	143211	157Gd	In-house
OX40	ACT35	158Gd	Fluidigm
CCR7	G043H7	159Tb	Fluidigm
CD28	CD28.2	160Gd	Fluidigm
CD45RO	UCHL1	161Dy	In-house
CD69	FN50	162Dy	Fluidigm
CRTH2	BM16	163Dy	Fluidigm
PD-1	EH12.1	164Dy	In-house
CD127	A019D5	165Ho	Fluidigm
CXCR5	RF8B2	166Er	In-house
CD27	L128	167Er	Fluidigm
DNAM-1	C1.7	168Er	In-house
CD45RA	HI100	169Tm	Fluidigm
CD3	UCHT1	170Er	Fluidigm
NKG2A	131411	171Yb	In-house
CD38	HIT2	172Yb	Fluidigm
TCF1*	7F11A10	173Yb	In-house
CD4	SK3	174Yb	Fluidigm
CXCR4	12G5	175Lu	Fluidigm
CD56	NCAM16.2	176Yb	Fluidigm
CD16	3G8	209Bi	Fluidigm

*Intracellular antibodies
