# Supplementary material for: Deep Phenotypic Analysis of Blood and Lymphoid T and NK Cells From HIV+ Controllers and ART-Suppressed Individuals
Source: Front Immunol. 2022 Jan 27;13:803417. doi: 10.3389/fimmu.2022.803417 (PMC8829545; doi:10.3389/fimmu.2022.803417)

Figure S1

A T Cell Gating Strategy

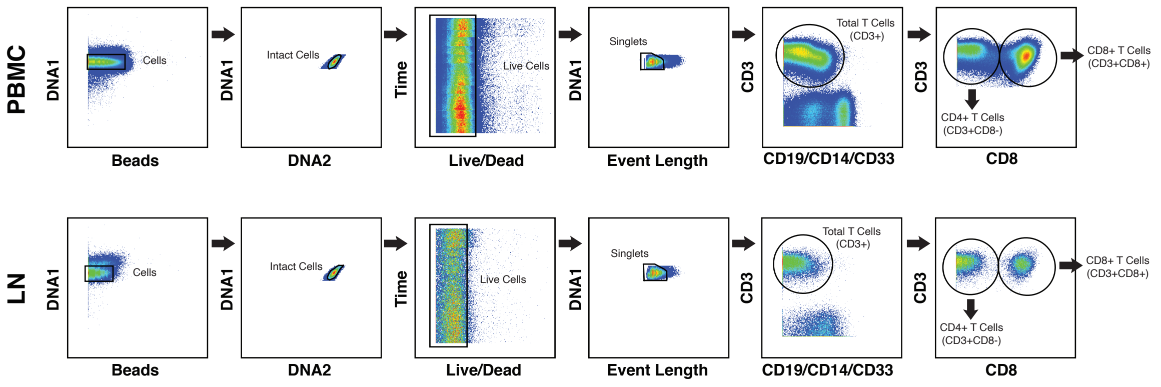

B NK Cell Gating Strategy

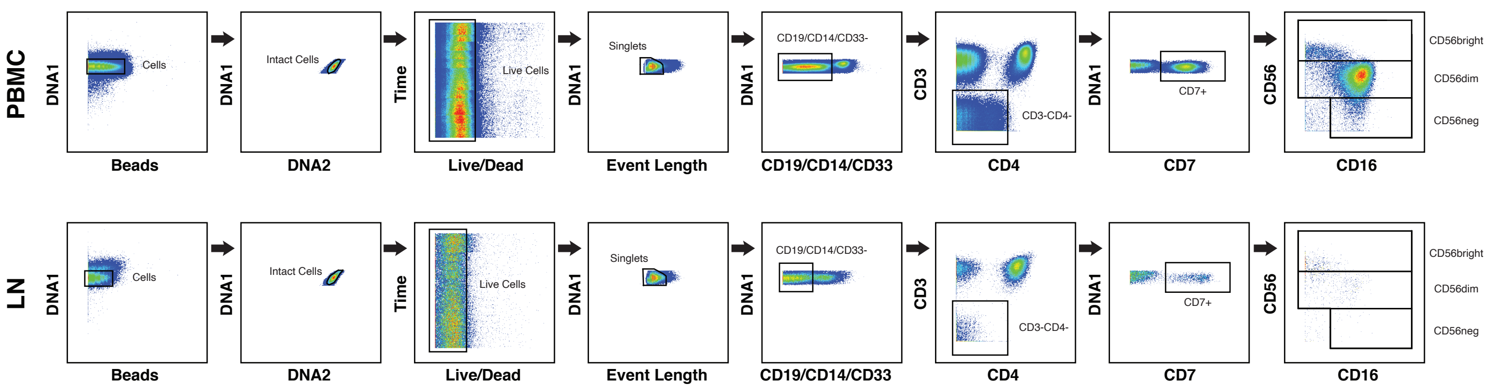

Figure S2

PBMC: CD3+ T Cells

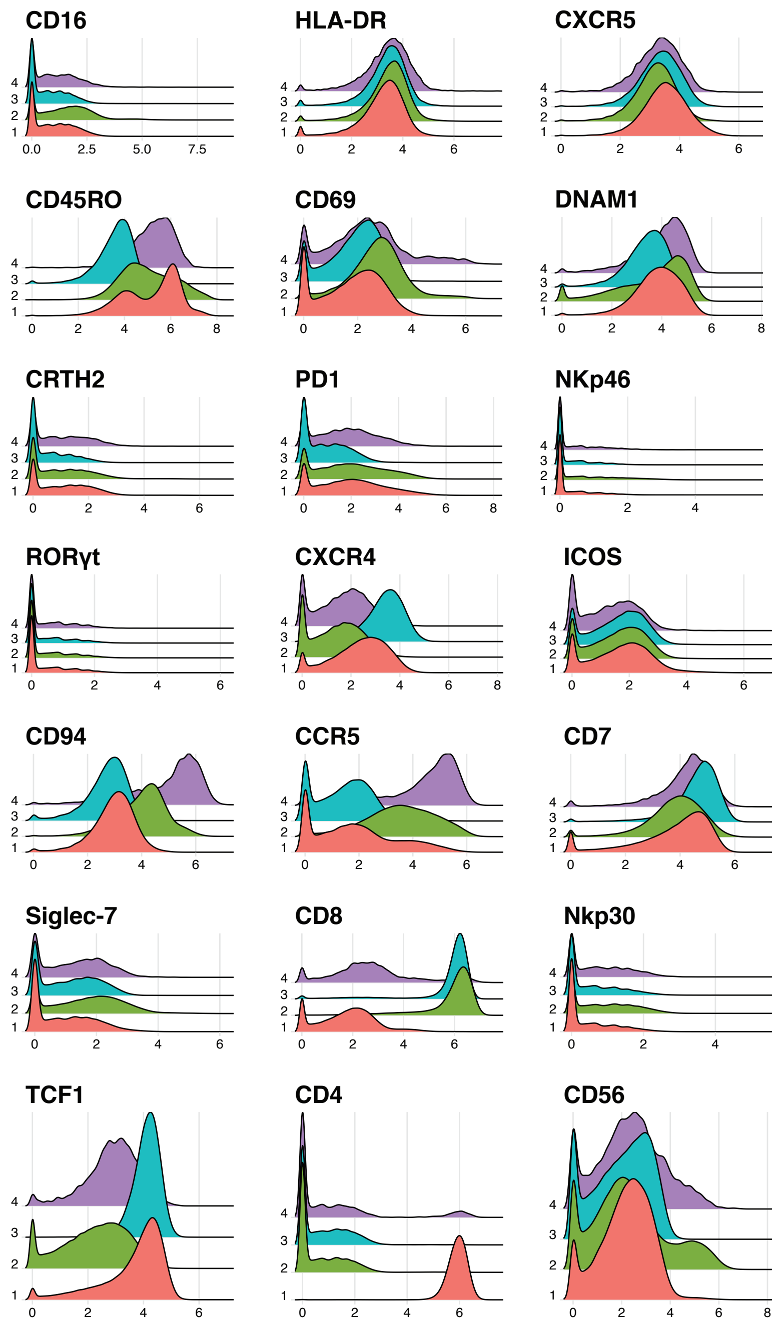

Figure S3

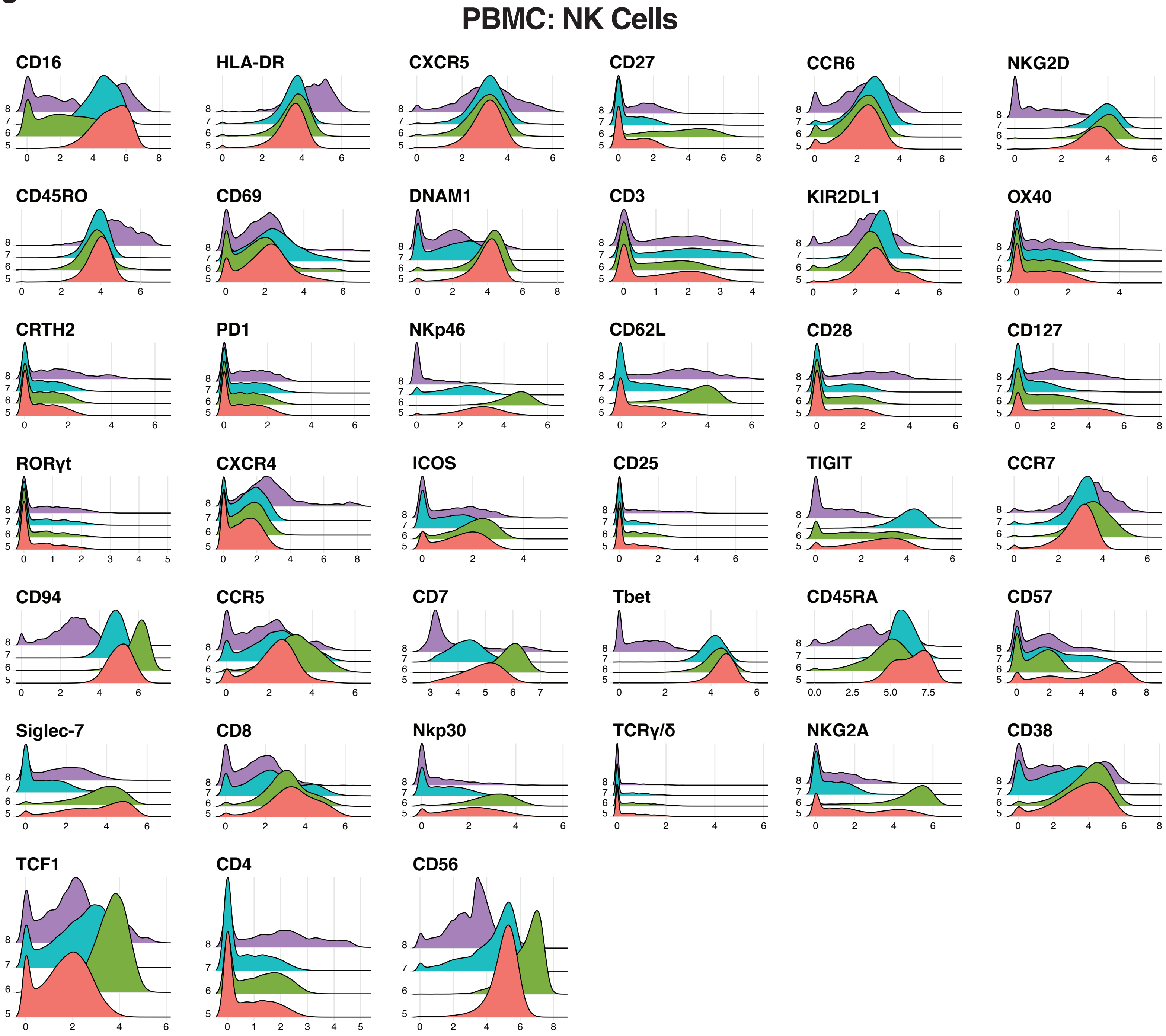
PBMC: NK Cells
CD16
HLA-DR
CXCR5
CD27
CCR6
NKG2D
CD45RO
CD69
DNAM1
CD3
KIR2DL1
OX40
CRTH2
PD1
NKp46
CD62L
CD28
CD127
RORγt
CXCR4
ICOS
CD25
TIGIT
CCR7
CD94
CCR5
CD7
Tbet
CD45RA
CD57
Siglec-7
CD8
Nkp30
TCRγ/δ
NKG2A
CD38
TCF1
CD4
CD56

Figure S4

LN: CD3+ T Cells

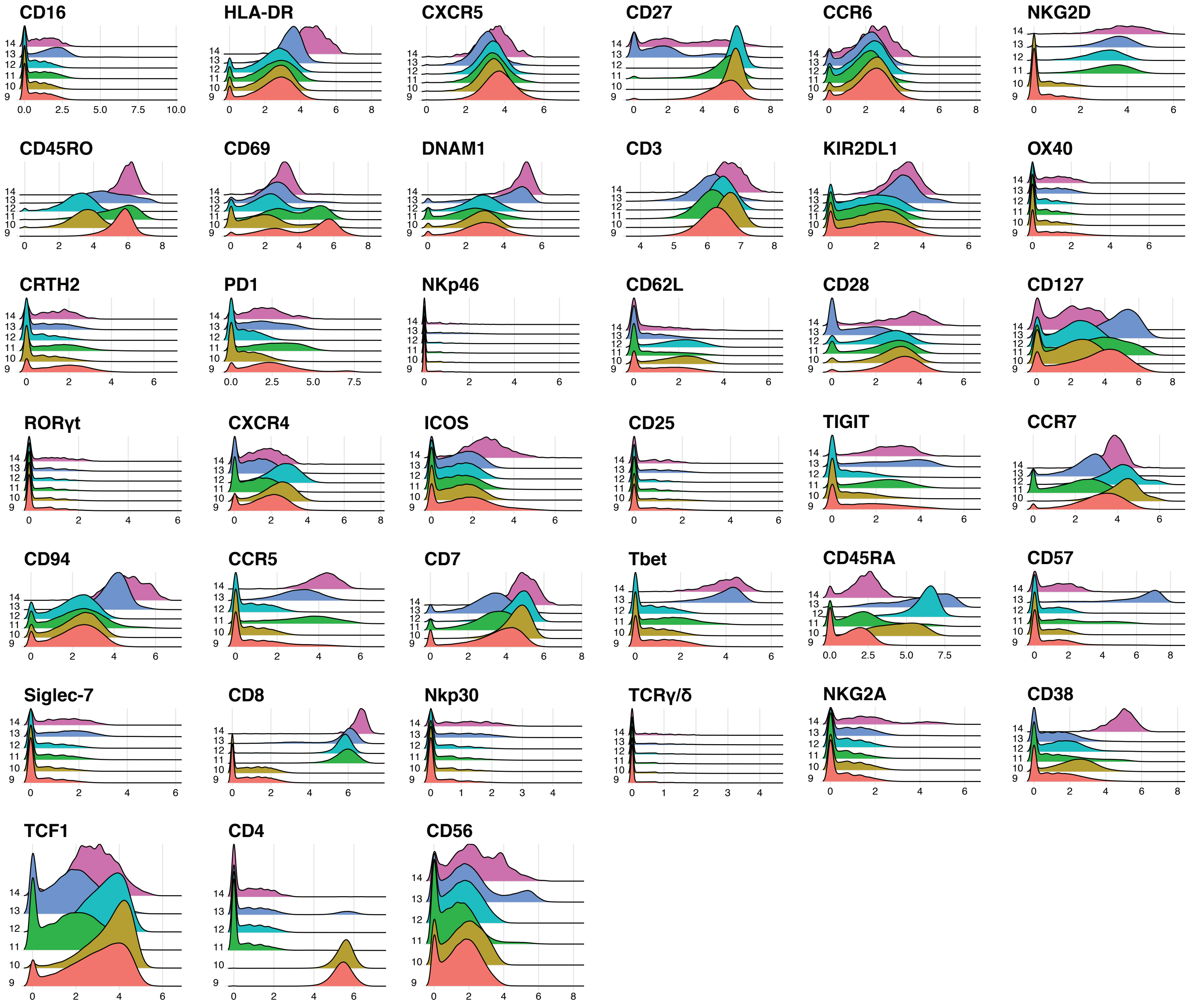

Figure S5

LN: NK Cells

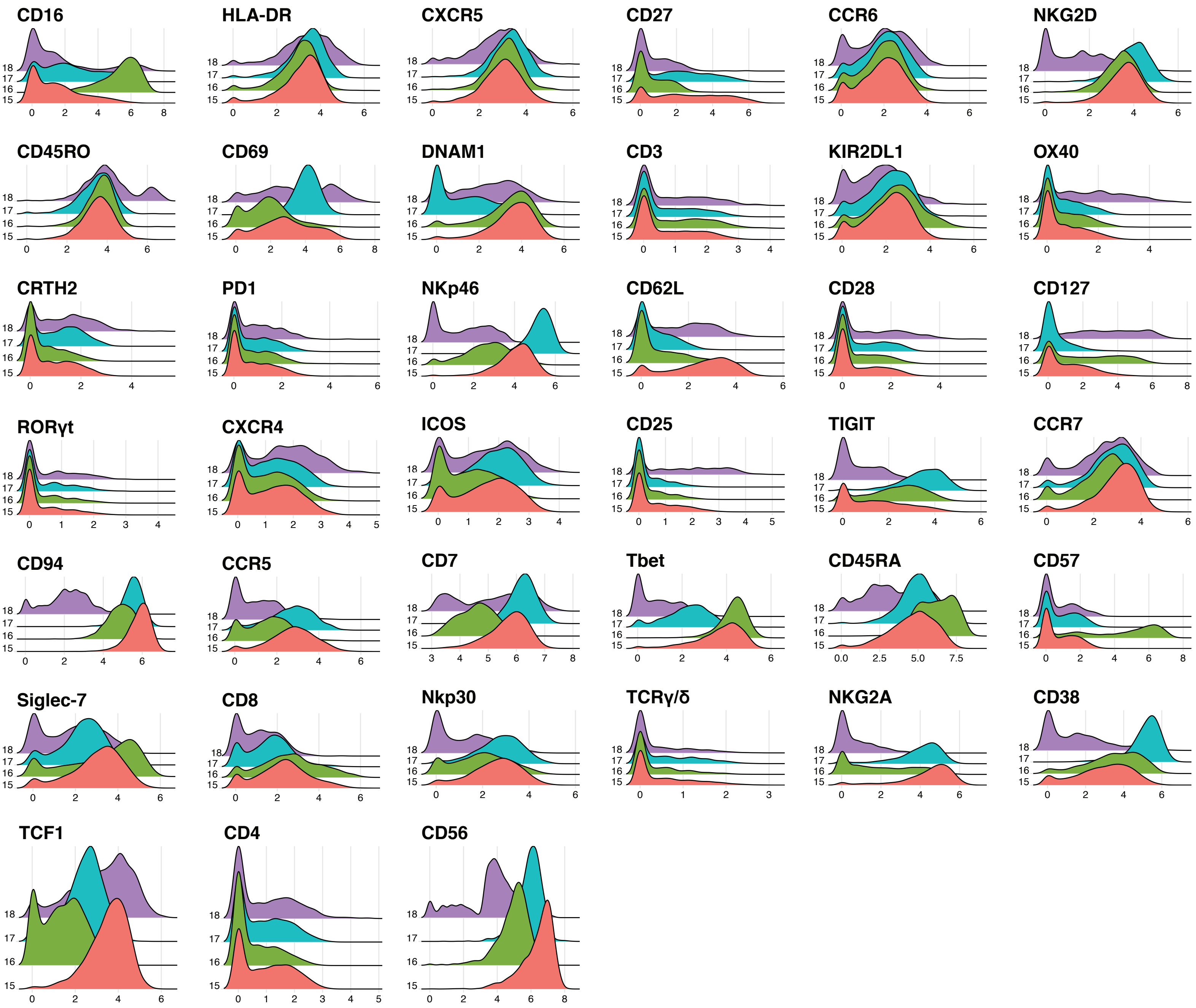

Figure S6

CD4+ T Cells Mean Signal Intensity

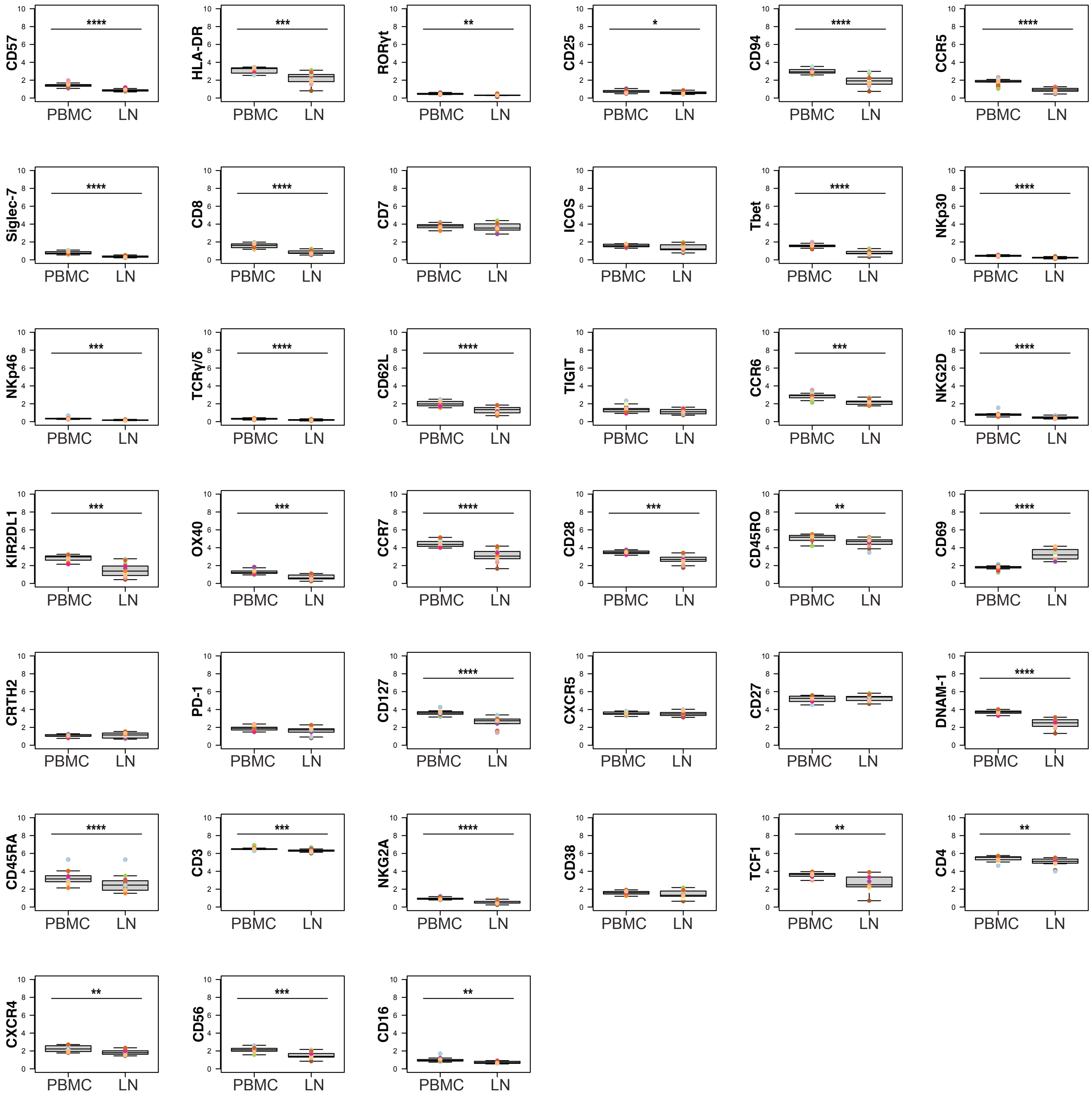

Figure S7

CD8+ T Cells: Mean Signal Intensity

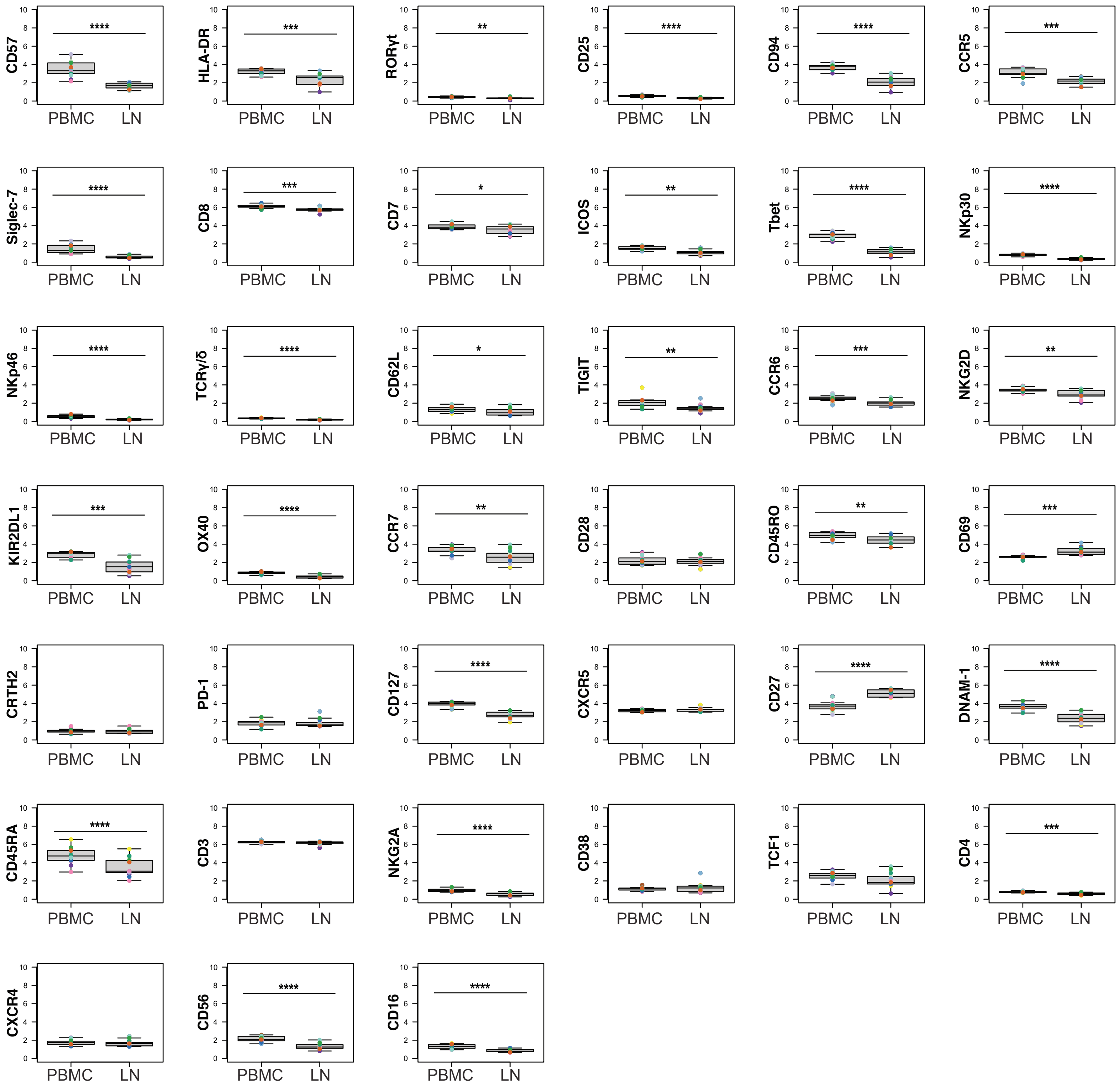

Figure S8

A Total NK Cells: Mean Signal Intensity

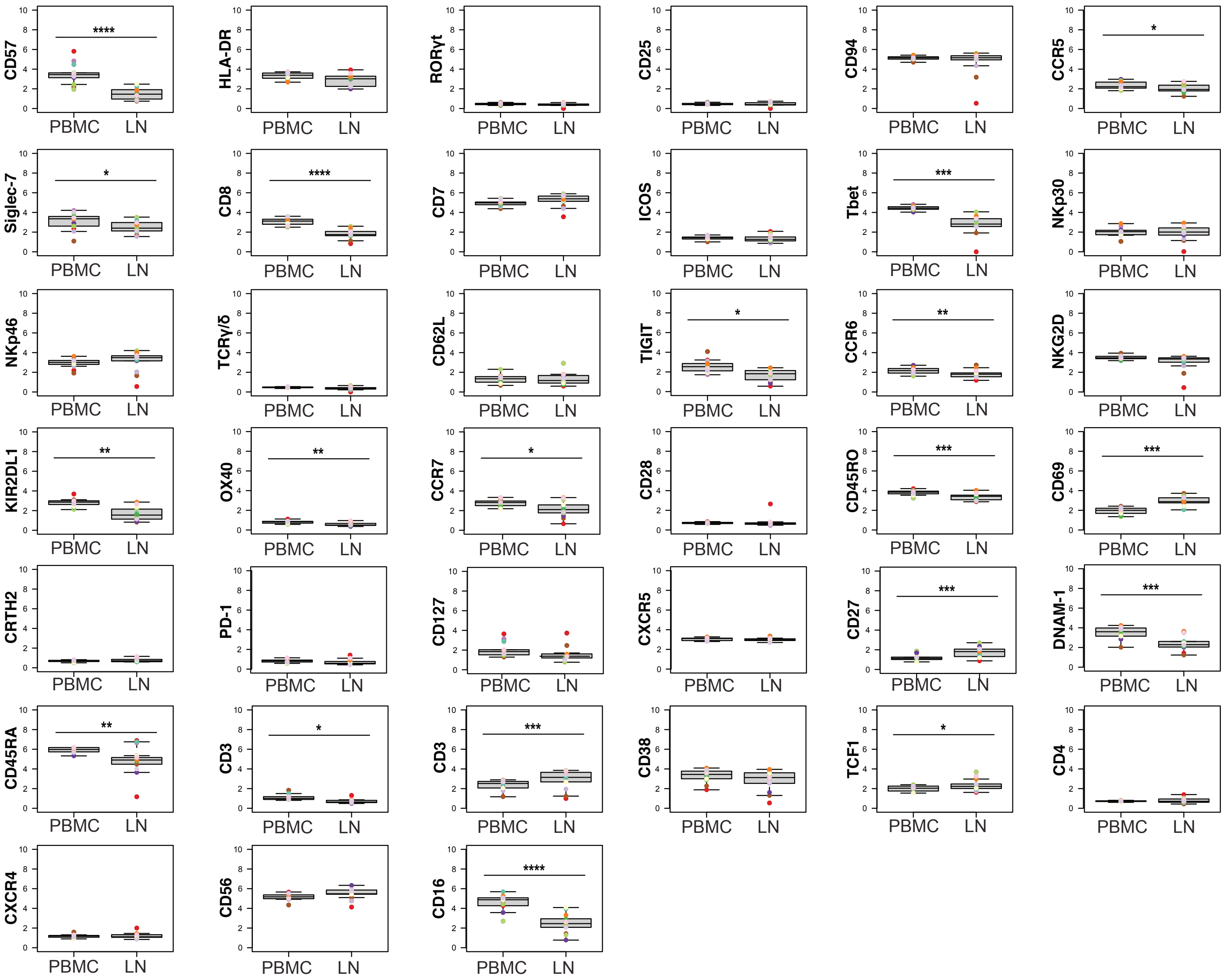

B PBMC

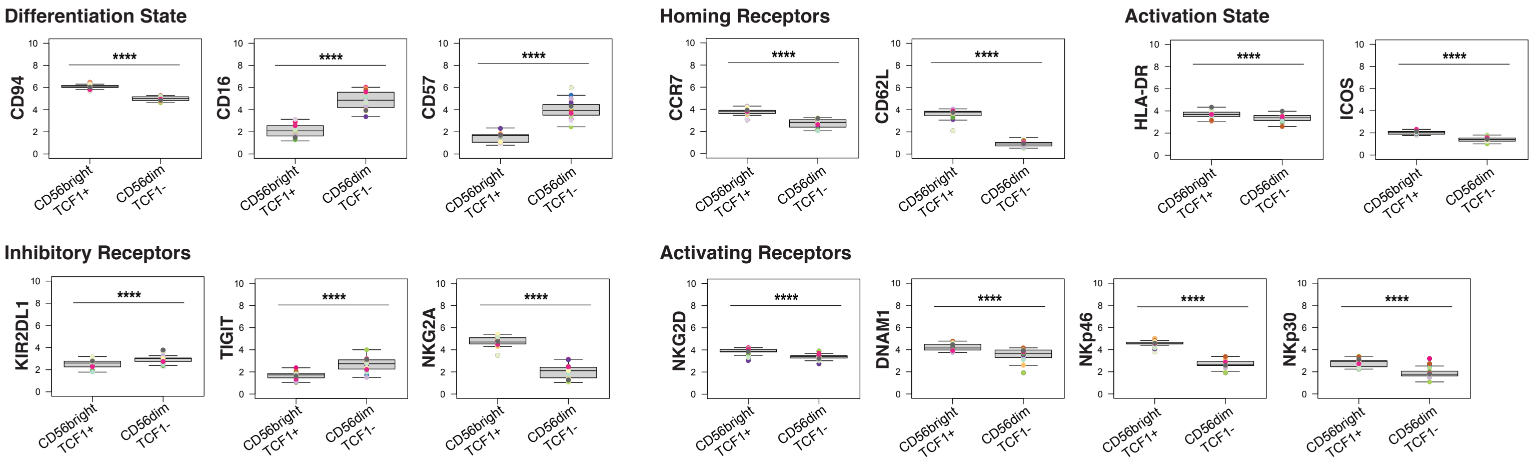

Supplement: Supplementary Figures 1 — CyTOF gating strategy to identify total T cells, CD4+ T cells, CD8+ T cells, and NK cells from PBMCs and LNs of PLWH. (A) Shown are gating strategies to identify live, singlet, total T cells, CD4+ T cells, and CD8+ T cells from a representative PBMC and LN specimen. (B) Shown are gating strategies to identify live, singlet NK cells from a representative PBMC and LN specimen. [file DataSheet_2.pdf]
